# Homeostatic Plasticity of Striatal Neurons Intrinsic Excitability following Dopamine Depletion

**DOI:** 10.1371/journal.pone.0006908

**Published:** 2009-09-04

**Authors:** Karima Azdad, Marcelo Chàvez, Patrick Don Bischop, Pim Wetzelaer, Bart Marescau, Peter Paul De Deyn, David Gall, Serge N. Schiffmann

**Affiliations:** 1 Laboratory of Neurophysiology, University of Brussels (ULB), Brussels, Belgium; 2 Department of Biomedical Sciences, Laboratory of Neurochemistry and Behaviour, Institute Born-Bunge, University of Antwerp, Wilrijk, Belgium; 3 European Graduate School of Neuroscience (EURON), Maastricht, The Netherlands; Baylor College of Medicine, United States of America

## Abstract

The striatum is the major input structure of basal ganglia and is involved in adaptive control of behaviour through the selection of relevant informations. Dopaminergic neurons that innervate striatum die in Parkinson disease, leading to inefficient adaptive behaviour. Neuronal activity of striatal medium spiny neurons (MSN) is modulated by dopamine receptors. Although dopamine signalling had received substantial attention, consequences of dopamine depletion on MSN intrinsic excitability remain unclear. Here we show, by performing perforated patch clamp recordings on brain slices, that dopamine depletion leads to an increase in MSN intrinsic excitability through the decrease of an inactivating A-type potassium current, *I*_A_. Despite the large decrease in their excitatory synaptic inputs determined by the decreased dendritic spines density and the increase in minimal current to evoke the first EPSP, this increase in intrinsic excitability resulted in an enhanced responsiveness to their remaining synapses, allowing them to fire similarly or more efficiently following input stimulation than in control condition. Therefore, this increase in intrinsic excitability through the regulation of *I*_A_ represents a form of homeostatic plasticity allowing neurons to compensate for perturbations in synaptic transmission and to promote stability in firing. The present observations show that this homeostatic ability to maintain firing rates within functional range also occurs in pathological conditions, allowing stabilizing neural computation within affected neuronal networks.

## Introduction

The striatum, the major input structure of basal ganglia, is involved in adaptive control of behaviour through the selection of behaviourally relevant information [1] and is thus important for the control of movements and the motivational, cognitive and emotional aspects of motor activity. The major population of striatal neurons, GABAergic striatal medium-size spiny neurons (MSN) receives massive innervation from the cerebral cortex, and cortical-like structures as hippocampus and amygdala, which exert a powerful glutamatergic excitatory influence. MSN occupy a strategic integrative position since these cortical inputs converge with dopaminergic afferents from the substantia nigra *pars compacta* and the ventral tegmental area that tightly regulate their activity. The involvement of this nigrostriatal dopaminergic pathway in Parkinson's disease, being subject to neurodegeneration, and in drug addiction, demonstrates its pivotal function in striatal physiology.

Dopamine has been shown to exert a variety of electrophysiological effects in MSN including the modulation of intrinsic conductances and the involvement in different types of corticostriatal synaptic plasticity [2], [3], [4]. However, despite series of available data [5], [6] the effects resulting from dopamine depletion on the MSN intrinsic excitability remain incompletely documented and puzzling. In addition, although the putative existence and the nature of a homeostatic response of these neurons in case of dopamine depletion have been recently suggested [7], they were never experimentally examined. This could be highly relevant since it has been demonstrated in a variety of physiological conditions such as memory storage or activity-dependent development that the average neuronal activity levels are maintained by homeostatic plasticity mechanisms that adjust synaptic strengths and/or intrinsic excitability to promote stability [8], [9], [10]. However, the existence of homeostatic plasticity and its nature in pathological conditions are mostly unknown.

Here, using perforated patch-clamp recordings, we investigated the alterations in MSN intrinsic excitability induced by dopamine depletion. Moreover, we correlated the changes in intrinsic excitability with modifications of the cortico-striatal excitatory synaptic transmission.

## Results

Recordings were performed on MSN that constitute the vast majority (90–95%) of striatal neurons and are easily identified in slice preparations by their size. These cells in control condition had resting membrane potentials of −74.17±0.69 mV (n = 22) and input resistances of 372.59±19.32 MΩ, parameters that are similar to those previously reported [11], [12], [13]. Less abundant classes of interneurons were readily identified based on their typical firing pattern (i.e cholinergic and fast spiking neurons) and excluded from the present study. Recordings were made using the perforated patch configuration in order to protect the integrity of the cytosol. This configuration allows, in contrast to the whole-cell configuration, to protect the integrity of the intracellular machinery and particularly the homeostasis of calcium and second messengers.

### Validation of the reserpine/alpha-methyl-*p*-tyrosine (AMPT) treatment induction of dopamine depletion in young rats

As stated in the introduction, although the consequences of dopamine receptors activation on striatal neurons physiology have been the subject of numerous studies, the alterations of MSN intrinsic excitability resulting from dopamine depletion remain incompletely documented. To address this question, we applied a dopamine depletion protocol easily and reliably usable on young rats (see methods). Before slice experiments were carried out, rats were treated with reserpine and AMPT using a regimen known to produce profound striatal dopamine depletion and parkinsonian motor symptoms. This model could be considered as strongly reliable since it has been previously shown to lead to similar consequences on striatal physiology than the 6- hydroxydopamine (6-OHDA) model [7], [14]. Nevertheless, as described below, the 6-OHDA model was also used to corroborate electrophysiological results.

We first determined the efficiency of this reserpine/AMPT treatment on weaned rat pups (P20 to P25). All animals used in this series and the subsequent series of experiments exhibit a profound cataleptic-like behaviour that is fully expressed in the two last days of treatment. We therefore examined by HPLC the degree of reserpine/AMPT-induced depletion in dopamine and its metabolites. The values obtained for dopamine, DOPAC and HVA in untreated and vehicle-treated animals were not significantly different (5873.4±954, 2486.6±420, 1233.6±119 ng/g tissue for untreated rats and 6454.3±858, 2273.9±484, 1270.4±125 ng/g tissue for vehicle-treated rats respectively; n = 5, p>0.05). As measured 5 hours after the last injection, the treatment with reserpine/AMPT led to a severe dopamine depletion of about 97% (100±16.24% for untreated animals, n = 5, 109.7±14.6% for vehicle-treated animals, n = 5 and 2.6±0.74% for reserpine/AMPT-treated animals, n = 6; p<0.001). The values obtained for dopamine metabolites revealed similar findings with a deficit of about 90% for DOPAC (100±16.9 % for untreated animals, n = 5, 91.4±19.5% for vehicle-treated animals, n = 5 and 8.6±3.6% for reserpine/AMPT-treated animals, n = 6; p<0.001) and a deficit of about 80% for HVA (100±9.7% for untreated animals, n = 5, 103±10.16% for vehicle-treated animals, n = 5 and 20.8±5.7% for reserpine/AMPT-treated animals, n = 6; p<0.001). To follow the time course of reserpine/AMPT treatment, we also examined the degree of reserpine/AMPT-induced depletion in dopamine and its metabolites 10 hours after the last injection. The values obtained for dopamine, DOPAC and HVA were strictly similar to those obtained 5 hours following the last reserpine injection with 3.5±1.28%, 3±0.25% and 14.8±1% of the control values for dopamine, DOPAC and HVA, respectively (n = 5, p<0.001).

**Figure 1:**
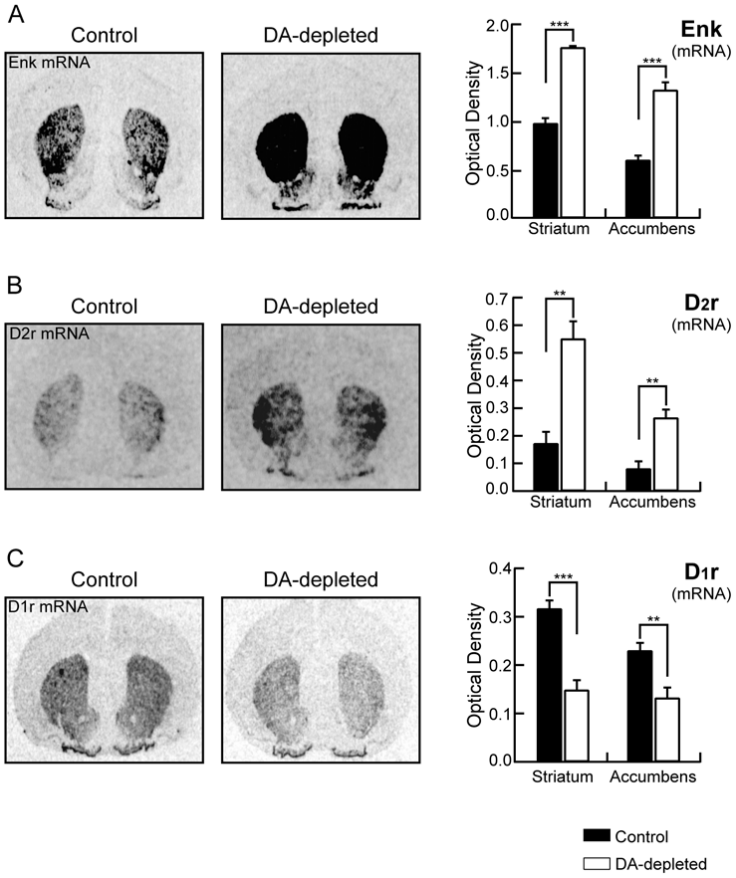
Dopamine depletion changes expression of specific striatal genes. *Left panels*, Autoradiograms generated by *in situ* hybridization using α-^35^S-labeled-labeled oligoprobes to identify enkephalin (A), dopamine D_2_ receptor (B) and dopamine D_1_ receptor (C) mRNA expression from untreated control and dopamine-depleted animals. *Right panels*, Levels of expression of enkephalin mRNA (A), D_2_ receptor mRNA (B) and D_1_ receptor mRNA (C) in striatum and nucleus accumbens in both conditions. (Untreated control rats n = 5, dopamine (DA)-depleted rats n = 6; data represent mean ± SEM expressed in optical density, arbitrary unit; **p<0.01, ***p<0.001).

In order to further investigate the effects of dopamine depletion on weaned rat pups and validate the functional efficiency of the model, expression of specific striatal genes as enkephalin, D_2_ receptor and D_1_ receptor, was studied by quantitative *in situ* hybridization histochemistry. This analysis showed a drastic increase of 80% in enkephalin mRNA expression in the dorsal striatum (0.964±0.0645 for control animals, n = 5, and 1.741±0.0126 for dopamine-depleted animals, AU, n = 6, p<0.0001; Fig. 1A) and of 120% in the nucleus accumbens (0.587±0.0531 for control animals, n = 5, and 1.304±0.0847 OD for dopamine-depleted animals, AU, n = 6, p<0.0001; Fig. 1A). D_2_ receptor mRNA expression presented similar changes with an increase of 220% in the dorsal striatum (0.172±0.0421 for control animals, n = 5, and 0.551±0.0628 for dopamine-depleted animals, AU, n = 6, p<0.001; Fig. 1B) and 227% in the nucleus accumbens (0.081±0.0264 control animal, n = 5, and 0.265±0.0297 for dopamine-depleted animals, AU, n = 6, p<0.001; Fig. 1B). In contrast, the expression of D_1_ receptor mRNA was modified in the opposite way by the reserpine/AMPT treatment. Indeed, a strong 53% decrease in D_1_ receptor mRNA expression was observed in the dorsal striatum (0.317±0.0165 for control animals, n = 5, and 0.148±0.0199 for dopamine-depleted animals, AU n = 6, p<0.0001; Fig. 1C) associated with a 42% decrease in the nucleus accumbens (0.230±0.0153 for control animals, AU, n = 5, and 0.132 ± 0.0215 for dopamine-depleted animals, AU, n = 6, p<0.01; Fig. 1C).

All these modifications in striatal gene expression are in perfect coherence with previous data reported in different models of dopamine depletion in rat and mouse [15 and references therein], validating therefore our protocol.

However, knowing that reserpine could also affect other monoaminergic system, to confirm our electrophysiological data (see below), we also unilaterally lesioned dopaminergic neurons in pups with the neurotoxin 6-OHDA as another widely accepted model of Parkinson disease. The quality of the lesion was validated in a pilot study by using tyrosine hydroxylase (TH) mRNA in situ hybridization on midbrain sections and dopamine transporter (DAT) autoradiography in the striatum showing a dramatic decrease in both markers (data not shown). It was further evaluated in midbrain from each recorded animal by TH mRNA in situ hybridization. This led to a 96.5±1.89 % decrease in TH mRNA in the substantia nigra pars compacta (n = 9, p<0,05, data not shown).

### Dopamine depletion increases the intrinsic excitability of medium spiny neurons

**Figure 2:**
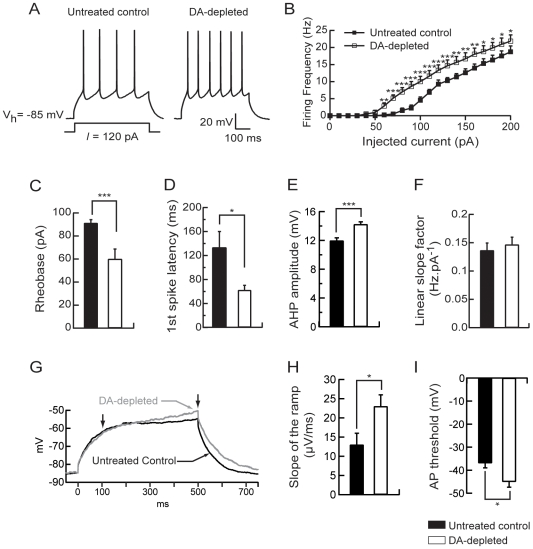
Dopamine depletion increases intrinsic excitability of medium spiny neurons. (A) Representatives traces showing that evoked action potentials (120 pA current pulse) were increased in dopamine-depleted MSN. (B) Summary plot of discharge frequency as a function of injected current illustrating the effect of dopamine depletion on the firing frequency. Dopamine depletion significantly shifted the curve to the left. (C) Histogram of the mean rheobase values showing the decrease of the average minimal depolarizing current amplitude required to elicit spike discharge (rheobase) in dopamine-depleted MSN compared to untreated controls. (D) Summary histogram of the mean first spike latency at the 120 pA current pulse illustrating the decrease of the first spike latency induced by dopamine depletion. (E) Histogram illustrating the effect of dopamine depletion on AHP amplitude at the 120 pA current pulse. (F) Histogram values of mean linear slope of the current-frequency plot in both conditions. (G) Representatives traces from untreated control and dopamine-depleted conditions, show the slowly developing ramp potential preceding firing. The slope of the ramp membrane potential was estimated between 100 and 500 ms after the induction of the 500 ms-depolarising step (arrows). (H) The increase of the slope of the ramp in dopamine-depleted condition is illustrated in the summary histogram. (I) Histogram values of the action potential threshold in dopamine-depleted MSN compared to untreated controls. (Untreated control medium spiny neurons n = 8, dopamine (DA)-depleted medium spiny neurons n = 8; data represent mean ± SEM; *p<0.05, **p<0.01, ***p<0.001).

To establish the functional consequence of dopamine depletion on intrinsic electrical properties of MSN, we have characterized the pattern of action potentials evoked in current-clamp recordings by depolarizing pulses applied through the recording pipette at the somatic level. A series of current pulses (500 ms) was delivered to a MSN at an interval of 10 s. These current pulses ranged from −50 pA to +200 pA in 10 pA steps. Using this protocol, our aim was to determine whether and how dopamine depletion directly modified the intrinsic excitability of these neurons. Electrical intrinsic properties of MSN were first investigated. The resting membrane potential in neurons from untreated control and dopamine-depleted rats was not significantly different (−74.17±0.69 mV, n = 22 vs −73.3±0.74 mV, n = 22, p>0.05), as the input resistance (372.59±19.32 MΩ, n = 22 vs 425.72±20.41 MΩ, n = 22, p>0.05) and the membrane capacitance (98.297±0.92 pF, n = 22 vs 98.364±2.93 pF, n = 22, p>0.05). Active cell membrane properties were evaluated by measuring the voltage response while injecting steps of depolarizing current of increasing intensities to construct current-frequency plots (Fig. 2B). The average minimal depolarizing current amplitude required to elicit spike discharge (rheobase) was significantly lower for MSN in dopamine-depleted animals (62.5±6.2 pA, n = 8) than the current to threshold for neurons in untreated control animals (91.25±2.95 pA, n = 8, p<0.05; Fig. 2C). As illustrated in fig. 2B, the action potential firing frequency increased with the intensity of the injected current. In neurons from dopamine-depleted rats, the current-firing frequency plot is shifted to the left with a firing frequency significantly increased for each current step (repeated-measures ANOVA, p<0.05; Fig. 2B) as shown for the representative 120 pA-evoked spike response (4±0.6 spikes for untreated control rats, n = 8 and 6.12±0.7 spikes for dopamine-depleted rats, n = 8, p<0.05; Fig. 2A). Moreover, the first spike latency in the 120 pA-evoked spike response was significantly decreased in MSN from dopamine-depleted rats (0.133±0.026 ms for control animals, n = 8, and 0.062±0.008 ms for dopamine-depleted animals, n = 8, p<0.05; Fig. 2D) and the AHP amplitude was slightly increased (11.96±0.39 mV for control animals, n = 8, and 14.23±0.34 mV for dopamine-depleted animals, n = 8, p<0.001; Fig. 2E). However, there was no modification of the linear slope factor of the current-frequency plots (0.139±0.143 Hz.pA^−1^ for untreated control rats, n = 8 and 0.146±0.0132 Hz.pA^−1^ for dopamine-depleted rats, n = 8, p>0.05; Fig. 2F).

*In vitro*, the MSN are also characterized by a slow ramp potential in response to depolarizing current pulses. To evaluate the effect of dopamine depletion on this parameter, we have measured the slope of the ramp potential defined by the membrane potentials at 100 and 500 ms at the subthreshold 500 ms depolarizing current pulse (Fig. 2G). The slope of the ramp potential was significantly higher for MSN in dopamine-depleted animals (0.023±0.003 mV/ms, n = 8) than the slope of the ramp potential for neurons in untreated control rats (0.013±0.003 mV/ms, n = 8, p<0.05; Fig. 2H). Moreover the action potential threshold was significantly decreased in MSN from dopamine-depleted rats (−36.92±2.03 mV for control animals, n = 8, and −45.02±2.4 mV for dopamine-depleted animals, n = 8, p<0.05; Fig. 2I).

**Figure 3:**
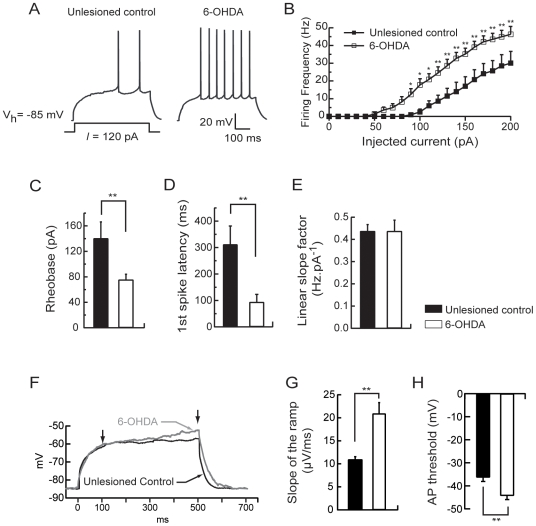
6-OHDA lesion-induced dopamine depletion increases intrinsic excitability of medium spiny neurons. (A) Representatives traces showing that evoked action potentials (120 pA current pulse) were increased in MSN from 6-OHDA lesioned animals. (B) Summary plot of discharge frequency as a function of injected current illustrating the effect of 6-OHDA lesion on the firing frequency. 6-OHDA lesion-induced dopamine depletion significantly shifted the curve to the left. (C) Histogram of the mean rheobase values showing the decrease of the average minimal depolarizing current amplitude required to elicit spike discharge (rheobase) in MSN from 6-OHDA lesioned animals compared to unlesioned controls. (D) Summary histogram of the mean first spike latency at the 120 pA current pulse illustrating the decrease of the first spike latency induced by 6-OHDA lesion. (E) Histogram values of mean linear slope of the current-frequency plot in both conditions. (F) Representatives traces from MSN in unlesioned control and 6-OHDA lesioned conditions, show the slowly developing ramp potential preceding firing. The slope of the ramp membrane potential was estimated between 100 and 500 ms after the induction of the 500 ms-depolarising step (arrows). (G) The increase of the slope of the ramp in 6-OHDA lesioned condition is illustrated in the summary histogram. (H) Histogram values of the action potential threshold in MSN from 6-OHDA lesioned animals compared to unlesioned controls. (medium spiny neurons from unlesioned control animals n = 7, medium spiny neurons from 6-OHDA lesioned animals n = 10; data represent mean ± SEM; *p<0.05, **p<0.01, ***p<0.001).

To determine whether these alterations in MSN excitability seen after reserpine treatment could be generalized to another widely accepted model of Parkinson disease, dopaminergic neurons were unilaterally lesioned with 6-OHDA. As in the reserpine-treated animals, the resting membrane potential (−81.48±0.5 mV, n = 7 vs −80.27±0.35 mV, n = 10, p>0.05) and the input resistance (215±22 MΩ, n = 7 vs 212.11±22.85 MΩ, n = 10, p>0.05) were not significantly different in neurons from unlesioned control and 6-OHDA lesioned rats. Active cell membrane properties were evaluated as described above. The current to threshold or rheobase was significantly lower for MSN in 6-OHDA lesioned animals (76±8.71 pA, n = 10) than for neurons in unlesioned control rats (141.43±26.13 pA, n = 7, p<0.01; Fig. 3C). In neurons from 6-OHDA lesioned rats, the current-firing frequency plot is shifted to the left with a firing frequency significantly increased for each current step (repeated-measures ANOVA, p<0.05; Fig. 3B) as for a representative 120 pA-evoked spike response (3.28±1.35 spikes for unlesioned control rats, n = 7 and 10.9±1.93 spikes for 6-OHDA lesioned rats, n = 10, p<0.01; Fig. 3A). The first spike latency in the 120 pA-evoked spike response was significantly decreased in MSN from 6-OHDA lesioned rats (0.313±0.07 ms for unlesioned control animals, n = 7, and 0.094±0.029 ms for 6-OHDA lesioned animals, n = 10, p<0.01; Fig. 3D). However, as in reserpine-treated animals, there was no modification of the linear slope factor of the current-frequency plots (0.44±0.03 Hz.pA^−1^ for unlesioned control rats, n = 7 and 0.44±0.05 Hz.pA^−1^ for 6-OHDA lesioned rats, n = 10, p>0.05; Fig. 3E).

Moreover, the slope of the ramp potential evoked by a subthreshold depolarizing current pulse was significantly higher for MSN in 6-OHDA lesioned animals (0.021±0.0023 mV/ms, n = 10) than the slope of the ramp potential for neurons in unlesioned control animals (0.011±0.0006 mV/ms, n = 7, p<0.05; Fig. 3F,G) and the action potential threshold was significantly decreased in MSN from 6-OHDA lesioned rats (−36.35±1.79 mV for unlesioned control animals, n = 7, and −44.29±1.7 mV for 6-OHDA lesioned animals, n = 10, p<0.01; Fig. 3H).

Altogether, these results show that the excitability of MSNs was identically modified in the reserpine-treated and 6-OHDA lesioned rats.

### Dopamine depletion alters the A-type potassium current in medium spiny neurons

**Figure 4:**
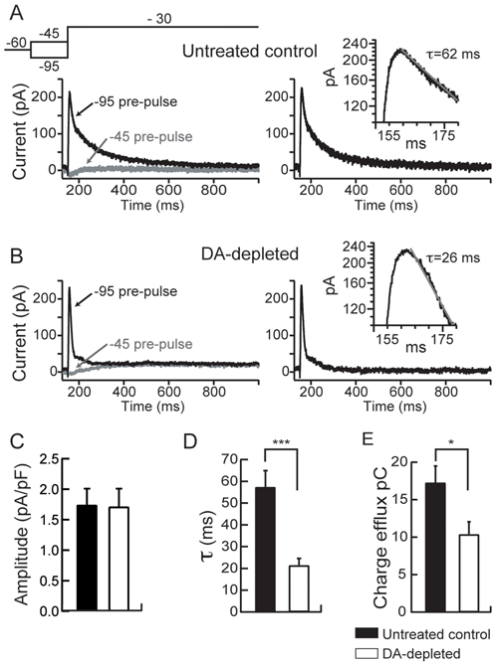
Dopamine depletion alters the A-type potassium current in MSN. (A, B) *Left column*, Representative current traces obtained in each studied condition (control and dopamine depletion) and evoked by the voltage-clamp protocol shown on the top. *Right column*, A-type currents obtained from subtraction of currents traces of the left column (see text for details). *Insets on the right column*, Semilogarithmic plots of A-type current traces show that the current inactivation could be fit by a monoexponential function. Summary histograms of the mean current amplitude density (C), the inactivation time constant (τ) (D) and the total charge efflux (E) in untreated control and dopamine depleted MSN. These histograms illustrate the significant decreases of the inactivation time constant (τ) and total charge efflux induced by dopamine depletion. (Untreated control medium spiny neurons n = 8, dopamine (DA)-depleted medium spiny neurons n = 8; data represent mean ± SEM; *p<0.05, ***p<0.001).

Voltage-gated potassium channels play a pivotal role in regulating neuronal excitability and are extremely diverse. Amongst these, major actors of the repetitive spiking and interspike interval regulation are the A-type potassium channels. More precisely, in MSN they sustained voltage-dependent inactivating A-type K^+^ current, *I*_A_, that controls the cell firing by slowing the rate of membrane depolarization [16], [17], [18], [19], [20]. Accordingly, pharmacological blockade of this current leads to an increased firing and shorten the latency to firing in MSN [21], [22]. Moreover, a D_2_ receptor-mediated regulation of this current in MSN has been suggested previously [21], [22]. We therefore asked whether the increase in MSN excitability in dopamine-depleted rats could also be sustained by a modification of the A-type K^+^ current. This current was therefore investigated in control and dopamine-depletion conditions as described. No significant modification in *I*_A_ amplitude could be observed in neurons from dopamine-depleted animals (Fig. 4B) as compared to neurons from untreated control rats (Fig. 4A), (1.74±0.27 pA/pF for untreated control animals, n = 7 and 1.71±0.3 pA/pF pA for dopamine-depleted animals, n = 7, p>0.05; Fig. 4C). However, the inactivation of this A-type K^+^ current was two times faster in neurons from dopamine-depleted animals (Fig. 4B) than that in the untreated control neurons (Fig. 4A) with significantly decreased inactivation time constants (τ) (52.21±7.66 ms for untreated control animals, n = 7 and 21.3±3.3 ms for dopamine-depleted animals, n = 7; p<0.001; Fig. 4D). As illustrated in Fig. 4A,B and quantified in Fig. 4E, such decrease in *I*_A_ inactivation time constant resulted in a dramatic decrease in charge efflux as determined by the area under current traces (17.24±2.25 pC for untreated control animals, n = 7 and 10.34±1.7 pC for dopamine-depleted rats, n = 7). Altogether, this strongly suggests that the decrease in *I*_A_ leads to the observed increase in intrinsic excitability of MSN in case of dopamine depletion.

### Effects of dopamine depletion on medium spiny neurons dendritic spines density and excitatory synaptic transmission

**Figure 5:**
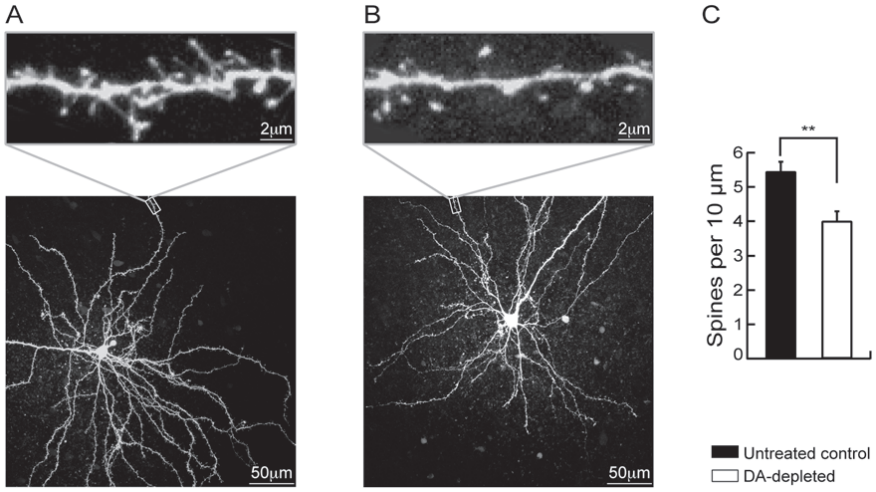
Dopamine depletion reduces spines density in MSN. Representative MSN in 280-µm-thick cortico-striatal slice from an untreated control rat (A) and a dopamine-depleted rat (B). Neurons were loaded with biocytin through the patch pipette and imaged by two-photon confocal microscopy. Maximum projection images of the soma and dendritic field (*bottom panels* A and B) and high-magnification projections of distal dendrite segments are shown (*top panels* A and B). (C) Histogram illustrating the effect of dopamine depletion on spine density. (Untreated control MSN n = 13, dopamine (DA)-depleted medium spiny neurons n = 7; data represent mean ± SEM; **p<0.01).

These results therefore point out to a major increase in intrinsic excitability and its underlying mechanism in MSN in case of dopamine depletion. However, in this condition, alterations in the cortico-striatal synaptic transmission have also been demonstrated with a decrease in asymmetric dendritic spines density [7], [23], [24] and a decrease in miniature EPSCs frequency [7] leading to the conclusion of a loss of glutamatergic synapses. This puzzling picture is a subject of debate for a while [7]. However, as suggested by Day et al. (2006), it could be argued that the increase in excitability may result from a homeostatic response of these neurons to the loss of excitatory synaptic inputs in case of dopamine depletion. To address this question, we examined both the dendritic spines density and the synaptic response in the same conditions as above. We have first analyzed the density of dendritic spines of MSN in dopamine-depleted and untreated control animals, by combining the patch-clamp technique and confocal microscopy. Intracellular loading of biocytin in individual MSN allowed its labelling without any overlapping dendrites from other neurons (Fig. 5A,B). At high magnification, dendritic spines could be identified and their density quantified (Fig. 5A,B, insets). Distal dendrites of MSN from dopamine-depleted animals and untreated control were examined (Fig. 5A,B, insets). Under these conditions, spine density was significantly decreased in reserpine/AMPT-treated animals (3.84±0.24 spines per 10 µm, n = 7) as compared to untreated control animals (5.44±0.28, n = 13, p<0.01; Fig. 5C). These data confirmed previous reports carried out by using different dopamine depletion protocols [7], [23], [24] and demonstrated again the decrease in excitatory inputs to MSN in this condition.

**Figure 6:**
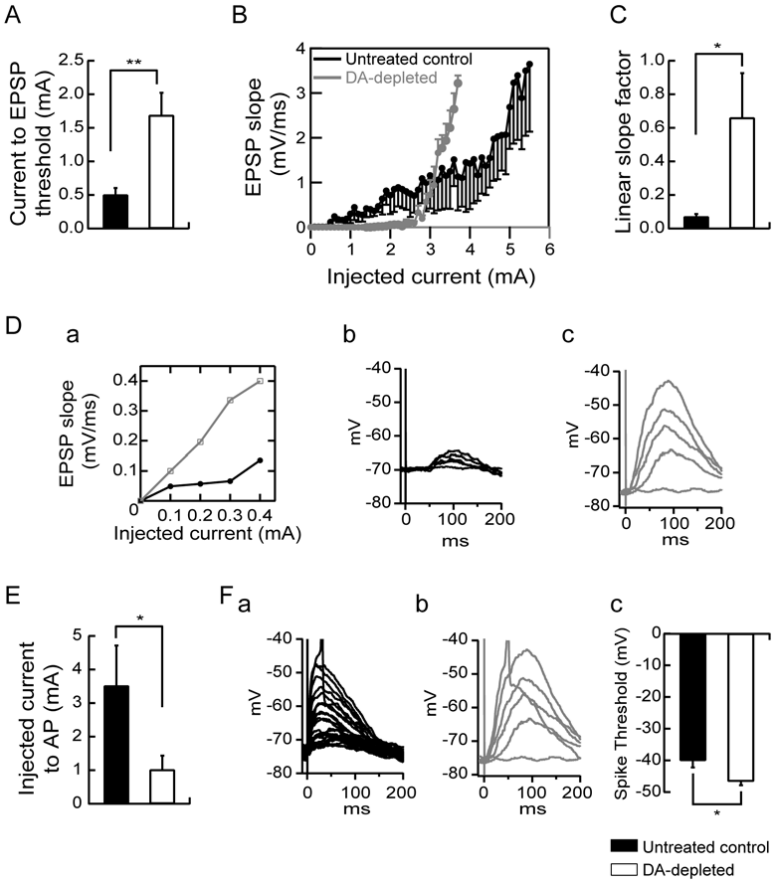
Dopamine depletion modulates excitatory synaptic transmission. (A) Summary histogram of the mean minimal stimulation threshold current required to evoke an EPSP showing a significant increase induced by dopamine depletion. (B) Summary plot of EPSP slope as a function of increasing intensities of stimulation from untreated control and dopamine-depleted MSN. (C) Histogram of the mean slope factor ((mV/ms).mA^−1^) of the linear part of stimulation-EPSP slope plots in both groups. Dopamine depletion induced an increase in the strength of the excitatory fiber-MSN synapse. (D) Representative stimulation-EPSP slope plots (a) and traces of stimulation-evoked EPSPs recorded in current clamp from untreated control (black in (a) and (b)) and dopamine-depleted (gray in (a) and (c)) MSN. (E) Summary histogram illustrating the intensity of stimulation current needed to evoke an action potential above threshold current to evoke an EPSP. The intensity of current to action potential was significantly decreased in neurons from dopamine-depleted animals which is shown in representative traces from untreated control (Fa) and dopamine-depleted (Fb) MSN. The spike threshold was decreased in dopamine-depleted MSN compared to untreated controls as shown in the representative traces (Fa, Fb) and the values histogram (Fc). (Untreated control medium spiny neurons n = 7, dopamine (DA)-depleted medium spiny neurons n = 7; data represent mean ± SEM; *p<0.05, **p<0.01).

The voltage changes induced in a postsynaptic neuron in response to synaptic activation, the evoked synaptic responses, are expected to depend not only on the amplitude, frequency and kinetics of synaptic currents, but also on the intrinsic membrane properties. Indeed, dendrites are endowed with a variety of voltage-dependent channels that may shape these synaptic responses, among which, a particularly important one is the A-type K^+^ current [25]. Moreover, since integration of synaptic inputs to produce an action potential is the fundamental step in neuronal computation, spiking activity representing the main neuronal output, we investigated both the strength of the excitatory synapses onto MSN from untreated control and dopamine-depleted rats and their ability to generate an action potential. To achieve this objective, a series of stimulations of increasing intensity (increment of 100 pA) was delivered to excitatory afferent fibers with an interval of 6 s between each stimulation. Comparison of stimulation-evoked EPSPs recorded in current clamp revealed several differences between untreated control and dopamine-depleted MSN. The minimal stimulation threshold current to evoke an EPSP is significantly increased in MSN from dopamine-depleted animals as compared to controls (0.5±0.1 mA for untreated control animals, n = 7 and 1.69±0.33 mA for dopamine-depleted animals, n = 7, p<0.01; Fig. 6A). Above these critical intensities of stimulation, EPSPs of increasing amplitudes were evoked and the slope of these EPSPs increased linearly with the intensity of the stimulation. The slope of EPSPs was measured and was used to construct stimulation-EPSP slope plots (Fig. 6B, Da). The evaluation of the slope factor of the linear part of stimulation-EPSP slope plots was used as a parameter of the strength of the excitatory fiber – MSN synapse (Fig. 6C, Da). Under these conditions, despite that the stimulation threshold current to evoke an EPSP is increased in dopamine-depleted animals, the initial linear slope factor of the stimulation-EPSP slope plots is significantly and dramatically increased in MSN from dopamine-depleted animals (0.66±0.26 mV.ms^−1^/mA, n = 7) as compared to untreated control animals (0.07±0.016, n = 7, p<0.05, Fig. 6C). This is clearly demonstrated by traces and stimulation-EPSP slope plots from representative neurons (Fig. 6Da,b,c) showing that once the threshold to evoke EPSP was reached, the increase in amplitudes and slopes of EPSP for each step of current was dramatically higher in MSN from dopamine-depleted animals (Fig. 6Da,c) than in neurons from controls rats (Fig. 6Da,b). As a consequence, above these threshold values of stimulation to induce an EPSP, the intensity of stimulation current needed to evoke an action potential was significantly decreased in neurons from dopamine-depleted animals (1.01±0.14 mA, n = 7) as compared to untreated control rats (3.51±1.2 mA, n = 7, p<0.05) (Fig. 6E and Fa,b for traces from representative neurons). Moreover, when suprathreshold EPSPs (Fig. 6Fa,b) were examined, it appeared that the spike threshold was also decreased in dopamine-depleted MSN (−46.63±1.21 mV, n = 7) as compared to untreated controls (−40.02±2.18 mV, n = 7, p<0.05) (Fig. 6Fc). Figure 6B clearly summarizes these data for the whole population of recorded neurons by showing that neurons from dopamine-depleted animals required a higher level of stimulation to evoke a first post-synaptic response whereas once it was attained, a lower level of stimulation was sufficient to reach the threshold for action potential initiation.

## Discussion

In the present study, we showed that dopamine depletion leads to an increase in MSN intrinsic excitability both in a reserpine-treatment induced dopamine depletion and in a 6-OHDA induced degeneration of dopaminergic neurons. We also showed that this increase in MSN intrinsic excitability takes place through the decrease in *I*_A_. We demonstrated that, despite the large decrease in their excitatory synaptic inputs, this increase in MSN intrinsic excitability resulted in an enhanced responsiveness to their remaining synapses, allowing them to fire similarly or even more efficiently following input stimulation than in control condition. Therefore, this increase in intrinsic excitability must be envisaged as a form of homeostatic plasticity demonstrated for the first time in case of dopamine depletion.

### Dopamine depletion–induced increase in intrinsic excitability relies upon *I*_A_ modulation

Despite available data on the effects of dopamine depletion on MSN [2], [3], [4], [5], [6], known consequences of dopamine depletion on their intrinsic excitability remain sparse and puzzling. Indeed, previous studies that have examined effects of dopamine depletion on MSN physiology have, in some cases, pointed out alterations of glutamatergic transmission with or without modifications of excitability [5], [6], [26], [27]. In both models of dopamine depletion used in the present study, the MSN intrinsic excitability was dramatically increased. In these conditions, we showed a decrease in the rheobase current, a decrease in the latency to the first spike, a leftward shift of the current – firing frequency plot, a decrease in the spike threshold and an increase in the slope of the ramp potential evoked by a subthreshold depolarizing current pulse. These results are consistent with some recent data from Fino et al. (2007) who showed, on acutely-depleted brain slices, an increase in the rheobase current and hence an increase in excitability. It is worth mentioning that resting membrane potential was reported as either unchanged [26], [27] or slightly but significantly altered [6].

We hypothesized that this increase in MSN intrinsic excitability could involve A-type potassium channels, which control neuronal excitability and repetitive firing [25]. The facilitation in cell depolarization in response to a subthreshold current pulse, which is strongly dependent on I_A_ [17], [18], is an additional argument involving this current in the increase in MSN excitability. Through isolation of *I*_A_, we demonstrated, that it was strongly modified in dopamine depletion condition, mostly by a speed up of its inactivation rate that led ultimately to a decrease in charge efflux. In MSN, pharmacological blockade of *I*_A_ leads to an increased firing rate and shortened latency to firing the first action potential [21], [22]. As same changes were detected in dopamine depletion, this strongly suggests a pivotal, if not exclusive, role of this current in the alterations in MSN intrinsic excitability observed in this condition. Moreover, this first direct demonstration of the involvement of *I*_A_ regulation in dopamine depletion strongly emphasizes the importance of this current in MSN intrinsic excitability. We also noted a slight but significant increase in AHP amplitude that would have lead, by itself, to a decrease in cell firing. However, a tight relationship between AHP amplitude and *I*_A_ has been reported showing that this latter current could indirectly affect, through modification in Ca^2+^ influx, the AHP amplitude in some populations of neurons [28].

### Dopamine depletion–induced increase in intrinsic excitability constitutes a form of homeostatic plasticity

Does this increase in intrinsic excitability constitutes a form of homeostatic plasticity as demonstrated in a variety of physiological conditions such as memory storage or activity-dependent development [8], [9], [10]? Indeed, in dopamine depletion condition, alterations in the cortico-striatal synaptic transmission have also been demonstrated with a decrease in asymmetric dendritic spines density [7], [23], [24] and a decrease in miniature EPSCs frequency [7] leading to the conclusion of a loss of glutamatergic synapses. Accordingly, we also demonstrated a decrease in dendritic spines density and established that the minimal stimulation threshold current to evoke an EPSP was dramatically increased, reinforcing the concept of decrease in glutamatergic inputs [7]. Therefore, the increase in excitability may result from a homeostatic response of MSN to the loss of excitatory synaptic inputs in case of dopamine depletion. This is not only important for the MSN somatic excitability, but also relevant for their dendritic responsiveness to excitatory inputs. Indeed, the voltage changes induced in a postsynaptic neuron in response to synaptic activation, depend not only on the amplitude, frequency and kinetics of synaptic currents, but also on intrinsic membrane properties. Dendrites are endowed with voltage-dependent channels and currents that shape synaptic responses, among which *I*_A_ is particularly important [25] and is strongly modified in case of dopamine depletion. *I*_A_ has been shown to limit EPSPs amplitude and accelerate the time course of their decay [25], [29]. Therefore, a decrease in this current should be expected to increase the dendritic responsiveness of these neurons and to increase the probability that EPSPs induced firing. That is exactly what we observed in case of dopamine depletion in MSN with a major increase in the linear slope of the stimulation - EPSP slope plots and a dramatic decrease of the stimulation current intensity needed to evoke the first action potential. Consequently, despite the major decrease in glutamatergic synaptic inputs, these modifications ultimately lead to a homeostatic restoration of a normal level of synaptic responsiveness. Moreover, they even lead to a higher responsiveness than in control condition. Indeed, the mean stimulation current intensity required to evoke the first action potential is lower in dopamine depletion than in control condition (see Fig. 6B). This latter observation could reconcile with some early reports that have proposed the existence of an increased excitatory synaptic cortico-striatal transmission [5], [26]. Although such homeostatic plasticity has readily been described in physiological conditions [8], [9], [10], it has rarely been deciphered in pathological conditions. Altogether, our observations fully establish the existence of a strong homeostatic mechanism in the pathological condition mimicking Parkinson disease, leading, despite their lower density of excitatory inputs, to a normal or even higher MSN synaptic and somatic responsiveness through a diminution of *I*_A_. Whether the molecular mechanism involves a modification of the phosphorylation state [30], [31] or membrane trafficking [32] of Kv4.2 channels, remains to be elucidated.

Neuronal networks are robustly able to compensate for perturbations in activity or synaptic transmission by homeostatic plasticity mechanisms, to ensure that firing rates are maintained within functional range, irrespective of which parameter is maladjusted. The present observations show that this ability is not only involved in physiological situations but also in pathological conditions allowing to stabilize, as far as possible, the neural computation within the affected neuronal network.

## Materials and Methods

### Animals and slice preparation

MSN were recorded in acute corticostriatal slices obtained from 20- to 25-day old Wistar rats (Iffa-Credo, Belgium). Animals were anaesthetized with halothane and killed by decapitation. The brain was quickly removed and placed in ice-cold (4°C) artificial cerebrospinal fluid (ACSF) saturated with 95% O_2_–5% CO_2_ and containing the following (in mM): 126 NaCl, 1.6 KCl, 1.2 NaH_2_PO_4_, 1 MgCl_2_, 2 CaCl_2_, 18 NaHCO_3_ and 11 glucose (pH 7.2–4, 290 mOsm/L) [21]. Coronal slices (280 µM thick) were cut in ice-cold ACSF using a vibratome (VT 1000S, Leica Instruments). Slices were incubated in ACSF (bubbled with 95% O_2_–5% CO_2_) at 32°C for at least one hour before recording. All procedures conformed with the standards of the Institutional Ethical Committee of the School of Medicine of the Université Libre de Bruxelles.

### Patch-clamp recording

Perforated patch-clamp recordings were performed on individual neurons identified by using infrared differential interference contrast microscopy (Axioskop 2FS, 40×/ 0.80w, Zeiss). Recording pipettes were pulled from borosilicate glass capillaries (Hilgenberg GmbH, Malsfeld, Germany) on a P-2000 pipette puller (Sutter Instruments, Novato, CA, USA) and presented resistances of 5–8 MΩ when filled with the patch pipette solutions containing the following (in mM): 80 K_2_SO_4_, 10 NaCl, 15 glucose, 5 HEPES, (pH 7.2–3 adjusted with KOH, 225–230 mOsm/l) and 100 µg/mL nystatin [33]. For experiments, slices were then transferred into a recording chamber where they were continuously perfused (2–3 mL/min) with oxygenated ACSF warmed to 32°C.

Passive cellular parameters were extracted in voltage clamp by analyzing current relaxation induced by a 10 mV hyperpolarized step from a holding potential of −80 mV as described previously [34]. In the perforated patch configuration, access resistance (*Ra*) was monitored to ensure that voltage attenuation in current clamp mode was always less than 10%. In addition, data from cells that showed >15% change in *Ra* were excluded from further analysis. All recordings were made with an Axopatch-200B amplifier (Axon Instruments) and acquired using the software Pulse (HEKA, Lambrecht-Pfalz, Germany) in combination with an ITC-16 AD/DA converter (Instrutech, NY, USA). Data were analyzed with Igor Pro software (WaveMetrics, Lake Oswego, OR, USA).

Action potential firing frequency was analyzed from current clamp recordings. Intrinsic excitability was investigated by setting membrane potential at −85 mV and injecting 500 msec step current pulses with 10 pA increments ranging from −50 to 200 pA. The sweep interval between each current step was of 10 sec. The oxygenated ACSF was supplemented with picrotoxin (25 µM, to block GABA_A_ receptors) and kynurenic acid (3 mM, to block AMPA and N-methyl-D-aspartate (NMDA) currents) to isolate the neuron from several major sources of neurotransmitters input whose release is known to be inhibited by dopamine [12], [35], [36]. The recordings were made in the fast current clamp mode and depolarization-evoked potential signals were filtered at a cutoff frequency of 2 kHz and subsequently digitized at 5 kHz. Each drug was applied for at least 6 min before starting the evoked action potentials protocol.

A-type potassium current recordings were pharmacologically isolated and recorded in the voltage-clamp mode by using an oxygenated extracellular solution containing the followings (in mM): 110 NaCl, 2.5 KCl, 1.2 NaH_2_PO_4_, 18 NaHCO_3,_ 11 glucose, 3 MgCl_2_, 30 TEA, 0.1 EGTA, 1 µM TTX and 25 µM picrotoxin (pH 7.2–4, 300 mOsm/L) and a perforated patch pipette solution containing the following (in mM): 120 K_2_SO_4_, 15 Glucose and 10 HEPES (pH 7.2–3 adjusted with KOH, 260–265 mOsm/l) and 100 µg/mL nystatin. Cells were clamped at −60 mV. K^+^ currents were evoked by stepping the membrane potential to −30 mV after a depolarized conditioning step (−45 mV) or a hyperpolarized conditioning step (−95 mV). By subtracting currents traces evoked by this protocol, the A-type K^+^ can be isolated [37], [38]. Current signals were filtered at 2 kHz and digitally sampled at 10 kHz.

To evoke synaptic excitatory potentials (EPSP), electrical stimulations were performed with a bipolar electrode (Phymep, Paris, France). To block inhibitory GABAergic input, recordings were performed in the presence of 25 µM picrotoxin (Sigma, St. Louis, MO). Current pulses with a duration of 150 µs were delivered in ascending order with 0.1 mA increments at 6 s intervals. These pulses were generated by an Iso-flex stimulus isolation unit (AMPI, Jerusalem, Israel) driven by a programmable Master 8 Stimulator/Pulse generator (AMPI) synchronized with data acquisition through Pulse software.

### Dopamine depletion

#### Reserpine/ alpha-methyl-p-tyrosine (AMPT) treatment

Dopamine was depleted by administrating reserpine and alpha-methyl-*p*-tyrosine (AMPT) intraperitoneally (i.p.). Animals in the reserpine/AMPT treatment received a first i.p. injection of 5 mg/kg reserpine in a 0.08% glacial acetic acid vehicle (0.9% saline solution) and 5 to 6 hours later an i.p. injection of 300 mg/kg AMPT in vehicle (0.9% saline solution) for 2 successive days [39]. The third day, 2 h prior experiments, animals received a last i.p. injection of reserpine. We made two groups of control animals, one group of untreated animals and one group of vehicle-treated animals. This last group received the same protocol as the reserpine/AMPT treated animals, with three days i.p. injection with 0.08% glacial acetic acid vehicle or vehicle alone corresponding respectively to reserpine or AMPT injections. All injections were given in a volume of 3 mL/kg.

#### 6-hydroxydopamine-induced lesion

Fifteen to 18-day old rats were anesthetized and placed on a stereotaxic frame and 20 µg of 6-OHDA (2 µl of 10 µg/µl dissolved in saline containing 0.2 mg/ml ascorbic acid) was injected into the right striatum with a blunt needle at the following coordinates (bregma as a reference) anterior +0.3 mm, lateral −3.0 mm, ventral +4.0 mm to the surface of the brain. The injection rate was 0.5 µl/min, and the syringe was left in place for 5 min before being withdrawn. All animals were pre-treated with desipramine (25 mg/kg, i,p., Sigma) 30 min before procedure to protect the noradrenergic neurons. Animals were anaesthetized with halothane and killed by decapitation 10 days after the injection and cortico-striatal slices were prepared as described above. Midbrain from each recorded animal was frozen for TH mRNA *in situ* hybridization (see below).

### Assessment of dopamine and dopamine metabolites levels

To determine the magnitude of dopamine depletion induced by combined administration of reserpine and AMPT, 5 h and 10 h after the last i.p. injection animals were decapitate and their brains rapidly removed and the striatum dissected on ice and frozen at −80°C. 7–10 mg of tissue was homogenized (30 sec, 5000 rpm) in 340 µL homogenization solution (0.1 M HCl + antioxidant mixture 0.1% Na_2_S_2_O_5_, 0.01% Na_2_EDTA) with an addition of 10 µL internal standard mixture containing 1024 ng/mL DHBA and 4096 ng/mL 5HNMT. The probe was washed in 350 µL homogenization solution, which was added to the first tube. After centrifugation (20800 g, 30 min, 4°C), 300 µL of the clear supernatant was mixed with 20 µL homogenization solution. After vortexing, the sample was diluted 1/4 with homogenization solution. This final dilution was filtered through a 0.2-µm-membrane filter (Ultrafree®-MC filter unit, Millipore Corporation, Bedford, MA, USA) and stored at −75°C until HPLC-ED analysis.

Chromatographic analysis was performed using ionic pair reversed chromatography on a BAS 200B HPLC system (Bioanalytical Systems, West Lafayette, IN, USA) equipped with a dual glassy carbon working electrode for electrochemical detection. The mobile phase, which was kept at a constant temperature of 35°C, consisted of a sodium phosphate-citrate buffer containing OSA (octane-1-sulfonic acid sodium salt monohydrate). Methanol was added as organic phase. Separation was performed on a microbore reversed-phase column (Hypersil, C18 BDS, 3 µM, 150 mm×1.0 mm I.D., LC Packings, Zurich, Switzerland). The use of a calibrated splitter system (Acurate™, LC Packings) allowed only 10% of the total pump flow-rate (400 µL/min) to pass over the analytical column, resulting in a flow of 40 µL/min. ChromGraph® Report software (Bioanalytical systems, West Lafayette, IN, USA) was used to analyze chromatographs and calculate compound concentrations by comparing ratios of each endogenous substance to the internal standard with linear calibration graphs. Detection limits (2× peak-to-peak noise of the baseline) were as follows: dopamine, 117 ng/g wet weight; DOPAC (3,4-dihydroxyphenylacetic acid) 79 ng/g wet weight; and HVA (homovanillic acid) 148 ng/g wet weight [40].

### *In situ* hybridization

Rats were killed by decapitation 2 to 3 h after the last injection. Their brains were quickly removed and frozen in 2-methylbutane cooled by dry ice. 18 µm-thick coronal sections were serially cut at the level of the striatum. The sections were thaw-mounted onto slides coated with poly-L-lysine. Mounted tissue sections were stored at −20°C until use. The sections were fixed in a buffered 4% formaldehyde solution freshly prepared from paraformaldehyde for 30 min and rinsed in 2X PBS 0.1 M. All sections were dehydrated and dipped for 5 min in chloroform. After air drying, the sections were incubated overnight at 42°C with 0.35×106 cpm per section of α-^35^S-labeled probes diluted in hybridization buffer, which consisted of 50% formamide, 4X SSC (1X SSC: 0.15M NaCl, 0.015M sodium citrate, pH 7.4), 1X Denhardt's solution (0.02% each of polyvinylpyrrolidone, bovine serum albumin, ficoll), 1% sarcosyl, 0.02M sodium phosphate, pH 7.4, 10% dextran sulfate, yeast tRNA at 500 µg/mL, salmon sperm DNA at 100 µg/mL, and 60 mM dithiothreitol. All reagents were obtained from Sigma (St. Louis, MO, USA). After hybridization, the sections were rinsed for 4×15 min in 1X SSC at 55°C, dehydrated, and covered with Hyperfilm-βmax film (Amersham, Buckinghamshire, UK) for 1 week (enkephalin) or 2 weeks (D_1_ receptor, D_2_ receptor, TH).

#### Oligonucleotide probes

The probes were synthesized on an Applied Biosystems (Foster City, CA) 381A DNA synthesizer with a GC to AT ratio between 45 and 65%. The enkephalin probe was complementary to nucleotides 4165–4199 (5′-GTGTGCATGCCAGGAAGTTGATGTCGCCGGGACGTACCAGGCGG-3′), the D_2_ probe to nucleotides 874–917 (5′-GGAGGAGTAGACCACAAAGGCAGGGTTGGCAATGATACACTCAT-3′), the D_1_ probe to nucleotides 1439–1483 (5′-GTGGTCTGGCAATTCTTGGCATGGACTGCTGCCCTCTCCAAGGC-3′) and the TH probe was 5′- GGCCAGGGTGTGCAGCTCATCCTGGACCCCCTCCAAGGAGCGCT-3′. Oligonucleotides were labeled with α-^35^S dATP (DuPont-NEN, Boston, MA, USA) at their 3′ end by terminal DNA deoxynucleotidylexotransferase (Gibco-BRL, Gaithersburg, MD) and purified with a nucleic acid purification cartridge (G50, Nensorb 20/DuPont-NEN).

#### Dopamine transporter autoradiography

DA transporter (DAT) binding was defined with 50pM [^125^I]RTI-55 (Perkin Elmer) in the presence of 1 µM fluoxetine (Sigma) in buffer A (Tris 50 mM, pH 7.4 ; NaCl 120 mM ; KCl 5 mM ; BSA 0.25%) . Nonspecific binding was determined with 10 mM GBR 12935 (Sigma). Following incubation for 1 h at room temperature, slides were washed (2X20 min) in icecold buffer, followed by one dip in ice-cold distilled water. Slides were dried under a gentle stream of cool air and then apposed for 24 h to Kodak Biomax MR film.

#### Data analysis

Digitalized images with 256 grey level were generated from the autoradiographs with the public domain NIH image 1.61 program (N.I.H., Bethesda, MA), a Power Macintosh G3, and a CCD video camera (Dage-MTI, Michigan City, IN) with fixed gain and black level. The digitalized images were analyzed on the ImageJ software (N.I.H., Bethesda, MA). Measurements were taken from the dorso-striatum and the nucleus accumbens. The optical density of film background was subtracted from each optical density (OD). Under appropriate conditions, the relative change in OD is a reliable indicator of changes in mRNA expression allowing for comparison of expression of specific mRNAs under different conditions.

### Confocal imaging

In a separate series of whole-cell recordings used for morphological reconstruction, the intracellular solution contained (in mM): 119 KMeSO_4_, 1 MgCl_2_, 0.1 CaCl_2_, 10 HEPES, 1 EGTA, 12 phosphocreatine, 2 Na_2_ATP, 0.7 Na_2_GTP, pH 7.2–3 adjusted with KOH, 280–300 mOsm/L and 0.4 % biocytin (Sigma-Aldrich, Bornem, Belgium). MSN were filled with biocytin during 15 minutes and fluorescence was subsequently revealed by cytochemistry. To this end, slices were fixed by immersion in 4% paraformaldehyde overnight. Biocytin was revealed with streptavidin-conjugated fluorescein isothiocyanate (FITC; Jackson Immunoresearch, UK) diluted 1∶200. After three rinses in TBS, slices were mounted on coverslips with «Slow Fade Light» anti-fade mounting medium (Invitrogen, Merelbeke, Belgium) in 50% glycerol and secured with nail polish. Images of the fluorescent cells were acquired using a LSM 510 META laser scanning confocal system (Zeiss, Oberkochen, Germany) mounted on an Axiovert 200M inverted microscope (Zeiss) equipped with c-Achroplan NIR 40×/0,8 W objective (Zeiss). The excitation beam of an Argon laser (488 nm) and band-pass emission filters (500–550 nm) were used for selective detection of the green fluorochrome. Sequential optical sections of 2048×2048 pixels were taken at 0.42 µm intervals along the z axis.

### Dendritic spines counts

Two-dimensional maximum projection reconstructions of images and quantification were done using ImageJ software (N.I.H., Bethesda, MA). For each cell, the threshold value defining the cell surface was set using the ISODATA algorithm implemented in ImageJ software (N.I.H., Bethesda, MA). For each condition, 7–13 neurons from at least two separate experiments were used, and 7–13 dendrites from each neuron were analyzed. The average width of dendrite (40–100 µm length) was evaluated by measuring the apparent area in z-projection and dividing by the length of the dendrite. Dendritic spines numbers per unit length were obtained by visually counting the spines on z-projections and dividing by the length of dendrites. To avoid any bias, data acquisition and analysis were performed in blind.

### Data analyses and statistics

Data were statistically compared with one-way analysis of variance (ANOVA) followed by a Bonferroni's post-hoc test or when appropriate, paired Student’s t-test. Significance was assessed at p<0.05. All data are reported as means ± SEM.

### Drugs and reagents

Appropriate drug stock solutions were made and diluted with ACSF just before application. All drugs were bath-applied. Drugs used were picrotoxin, kynurenic acid.
